# Species in ecosystems and all that jazz

**DOI:** 10.1371/journal.pbio.2006285

**Published:** 2018-07-13

**Authors:** Oswald J. Schmitz

**Affiliations:** School of Forestry and Environmental Studies, Yale University, New Haven, Connecticut, United States of America

## Abstract

Ecosystem ecologists explore how different kinds of species fit together to drive ecosystem processes such as nutrient cycling and productivity. This research is motivated by theories that assume that the suite of traits that characterize a species’ form determines its function, that these traits have become fixed over evolutionary time, and that ensuing ecosystem process are not resilient to environmental change. Here, I explore new research that re-evaluates this theory. Recent results suggest that functional traits are malleable, enabling species to rapidly respond and adapt to each other as environmental conditions change with predictable effects on ecosystem processes. These basic research findings suggest that species adaptations may impart in ecosystems an inherent capacity to weather environmental changes, thereby offering deeper understanding about which biological attributes protect ecological functions and which are needed to restore damaged ecosystems.

Nature has been likened to a symphony orchestra in which individual species each are the different instruments that together produce a grand harmony [1,2]. Captured by this conception of nature, ecologists have painstakingly worked to understand how this harmony comes about. We categorize different species according to common properties such as their functional forms [3,4], describe the variety within each functional category and how it evolved [4,5], and, most importantly, conduct experiments to figure out how different functional forms of species complement one another to produce the ensuing ecosystem functions [2,6]. It is akin to assembling various instruments into different orchestral configurations to see how they play together and produce their harmonies. And the effort has yielded some surprising findings that are overturning long-standing conceptions about the way ecosystems function.

When I began my research 25 years ago, it was widely understood that ecosystem functioning basically results from species together producing, exchanging, and recycling nutrients and energy [7]. Fundamentally, plants take up carbon dioxide, nutrients, and water to produce edible tissue; herbivores eat plant tissues and assimilate the embodied energy and nutrients; and predators do the same by eating herbivores. Plant and animal waste material and dead individuals are in turn broken down by decomposers into their constituent chemical elements. These released elements are then recycled, driving new plant production, and so forth.

It was theorized at the time that such ecosystem functioning was tightly orchestrated. Consigned by their evolved functional forms, species performed according to a set musical score. Moreover, in nature’s orchestra, plants—or rather their ability to produce edible, nutritious tissues—provided the lead melody, and all other species provided the accompaniment. But decades of research by my lab group led me to realize that these long-held theories were incorrect. We have instead discovered that there may not be a lead at all, and, more startlingly, that there may not even be a set musical score that is being followed. Instead, the harmony seems to emerge from an improvisation in which the different instruments play off of one another, each reacting and adapting to the others’ changes in the music they produce. Nature, it seems, is more like a jazz ensemble than a symphony orchestra. Moreover, our research has revealed some amazing capacities of species to react and adapt in response to changes in their community.

We made our discoveries by studying interactions among species that live naturally in meadows, including predatory spiders, grasshopper herbivores that are preyed upon by the spiders, and grass and herb plants that are eaten by the grasshoppers. They are ideal groups of species to study because we can experimentally assemble different combinations and house them within mesocosm cages within their natural field setting, and modern electronic instruments allow us to accurately trace the flow of nutrients and measure rates of production and nutrient recycling [8]. In essence, we create miniature experimental ecosystems in the field to get a detailed, realistic look at how nature works.

We have found that in the absence of predators, grasshoppers prefer to consume nitrogen (N)-rich grasses to maximize their survival and reproduction. But when they face their spider predator, they adapt by shifting their habitat usage and diet in favor of herbs. Leafy herbs provide refuge habitat, allowing grasshoppers to evade predators. Moreover, chronic stress from fear of predation heightens grasshopper metabolism and agility, in turn inducing physiological changes that demand more carbon (C)-rich energy. Herbs contain more energy than grasses, hence the reason for the grasshopper diet shift [9]. Predator-caused changes in grasshopper foraging in turn drive changes in the fate and balance of energy and nutrients within the ecosystem, ultimately altering the rate at which plant and animal matter decompose and the amount of embodied elemental C and N that is recycled [9].

But this outcome only happened when grasshoppers face species of ambush predators that lie in wait in fixed locations within the vegetation. Grasshoppers don’t know exactly where these predators lurk, hence the risk. Nevertheless, they perceive the predators’ presence and so remain continuously vigilant. Grasshoppers change their behavior altogether when they face actively hunting predators who roam widely. Individual grasshoppers will encounter such a predator infrequently. It would thus be energetically inefficient for grasshoppers to be chronically stressed when living in ecosystems with active hunting predators. Grasshoppers adapt to this change by responding to imminent threat only upon encountering the roaming predator. In ecosystems with just active hunting predators present, grasshoppers prefer grasses to herbs, which concomitantly changes nutrient cycling within the ecosystem relative to when they faced ambush predators. Thus, while plants provide the nutrients to begin with, the functional nature of predators—whether they are ambush or widely roaming predators—has a strong hand in changing the functional nature of grasshoppers through changes in grasshopper behavioral and physiological traits and therefore nutrient demand. Hence, it is through species responding and adapting to one another that determines the fate of nutrients within the ecosystem.

These discoveries have caused us to change how we think about the link between species and ecosystems. The traditional view of a tightly orchestrated symphony holds that species have fixed, evolved functional forms—traits—that predetermine their fate, which in turn would make ecosystems fragile to any environmental change. In this view, change is expected to cause species traits to be maladaptive, leading to the demise of species along with loss of sustainability of ecosystem functioning. The new view suggests alternatively that species have considerable scope and capacity to adapt to each other and their environments and thereby may impart far more resilience to environmental stressors and disturbances that was once thought. When they are pushed hard enough, we further identify limits in their capacities to adapt and perform [10].

The physiological machinery enabling animals to adaptively respond to stress from perceived predation risk is highly conserved evolutionarily [11]. Hence, changes in nutrient balance within species and ecosystems should be widespread as prey species respond adaptively to their predators and resources [12]. Emerging research is showing this to be a promising new way to look at functioning in a variety of ecosystems [13,14].

But if species continually respond and adapt to each other depending on local environmental conditions, how do we ever hope to develop general scientific principles about how nature works? Here again, the new insights teach us that we must change how we conduct experimental research. It requires adopting a relational approach [6] in which a general understanding of how species improvise requires the use of transplant experiments that measure how their functional roles in different environmental contexts is related to changes in the expression of their traits. Examining how and why the same organisms express their traits in different ways in different environmental contexts opens the door to deeper understanding of ecosystems. Through this retooled approach we can identify different kinds of fundamental principles—principles grounded in the recognition that species’ trait responses and adaptations themselves change. As we document these changes, we can link them to ecosystem functioning under those same conditions [10]. With time, the insights gained from experimental ecosystems will provide a general understanding of not only how species interact to drive ecosystem functioning, but also what we can do to restore nature’s harmony by returning species who’ve lost the ability to play their part.

The ultimate message coming from my science is that species are not merely entities that we should describe, catalog, and admire for our passing enjoyment. Rather, plant and animal species alike have an amazing variety of functional forms that together instrumentally determine the sustainability and resilience of ecological systems that provide our life support. One should be awed by the amazing capacity of species to adapt to each other and their changing fortunes as they go about their business of living. And so I feel it is incumbent upon scientists not just to report these findings within our immediate professional circles but rather convey the findings and their importance to a broader public. My own effort in this regard is through writing books that present the science in accessible ways, telling stories about how I as a person relate to the natural world and the inspiration that I as a scientist get from studying nature [15]. My hope is that society will become similarly awed by and enthused about all of nature’s jazz. I believe that it is through greater mutual understanding and relatable appreciation of scientific discoveries that we can inspire society to begin admiring, respecting, learning from, and protecting all of the living species and the amazing ways they adapt and thrive to sustain the functioning of our shared planet.
